# Correction: Suppression of local type I interferon by gut microbiota–derived butyrate impairs antitumor effects of ionizing radiation

**DOI:** 10.1084/jem.2020191509032025c

**Published:** 2025-09-19

**Authors:** Kaiting Yang, Yuzhu Hou, Yuan Zhang, Hua Liang, Anukriti Sharma, Wenxin Zheng, Liangliang Wang, Rolando Torres, Ken Tatebe, Steven J. Chmura, Sean P. Pitroda, Jack A. Gilbert, Yang-Xin Fu, Ralph R. Weichselbaum

Vol. 218, No. 3 | https://doi.org/10.1084/jem.20201915 | January 26, 2021

The authors regret that, during the revision process, the Materials and methods section was not updated to reflect the final results used for publication, which involved the use of vancomycin or gentamicin, not a cocktail of antibiotics. The original and rewritten Antibiotics and bacteria administration section appear below. The error appears in print and in PDFs downloaded before September 17, 2025.

## Materials and methods

Original:

### Antibiotics and bacteria administration

For antibiotic administration, mice were orally fed with antibiotic suspensions (0.5 mg/ml ampicillin, 0.5 mg/ml gentamicin, 0.5 mg/ml metronidazole, 0.5 mg/ml neomycin, and 0.25 mg/ml vancomycin dissolved in autoclaved water) from 3 wk before tumor inoculation to experimental endpoints. Antibiotics were purchased from Sigma-Aldrich. For bacteria oral administration, GF C57BL/6 mice were gavaged weekly with 10^8^ CFU of *Lachnospiraceae* family member *K. alysoides* (TSD-26; purchased from ATCC and cultured with trypticase soya broth + 5% [vol/vol] defibrinated sheep blood in an anaerobic chamber) in 500 µl water per mouse from 3 wk before tumor inoculation to experiment endpoints.

Corrected:

### Antibiotics and bacteria administration

For selective antibiotic administration, mice received gentamicin (1 mg/ml) or vancomycin (0.5 mg/ml) in autoclaved drinking water for 1 mo. During the first week, the antibiotic water was also delivered by oral gavage (500 µl per mouse once daily) to ensure rapid acclimation. Control animals received autoclaved water without antibiotic for the same period. Tumor cells were inoculated on the day following the 1-mo antibiotic treatment, and mice remained on their respective water until experimental endpoints. For bacteria oral administration, GF C57BL/6 mice received 10^8^ CFU of *Lachnospiraceae* family member *K. alysoides* (TSD-26; purchased from ATCC) in 500 µl sterile anaerobic water by oral gavage once per week, starting 1 mo before tumor inoculation and continuing until experimental endpoints. The bacteria were cultured with trypticase soya broth + 5% [vol/vol] defibrinated sheep blood in an anaerobic chamber to mid-log phase, washed once and quantified by serial dilution plating. The inoculum was prepared fresh for each gavage, kept on ice, and delivered within 30 min. Control mice received an equal volume of sterile anaerobic water on the same schedule.

